# The Paracrine Effect of Hypoxic and Normoxic Cancer Secretion on the Proliferation of Brain Endothelial Cells (bEnd.3)

**DOI:** 10.3390/cells11071197

**Published:** 2022-04-02

**Authors:** Mariam Rado, David Fisher

**Affiliations:** Medical Bioscience Department, Faculty of Natural Sciences, University of the Western Cape, Robert Sobukwe Road, Bellville 7335, South Africa; 3580480@myuwc.ac.za

**Keywords:** cell division, cell proliferation, brain endothelial cells, cancer secretion, hypoxia, normoxia, U-87 cells, MCF7 cells, bEnd.3 cells

## Abstract

Background: This study aimed to investigate the disruption of cell cycle phases of bEnd.3 cells exposed to cancer paracrine secretion. Cancer cells have been reported to use the secretion of paracrine factors to compromise the endothelial barrier to prepare for their passage into the parenchyma. As cancer cells are known to act differently under conditions of hypoxia, we investigated how conditional media (CM) derived from breast and glioblastoma cells incubated under conditions of normoxia and hypoxia would affect proliferation of brain endothelial cells (bEnd.3). Methods: Brain endothelial cells (bEnd.3) were cultivated with normoxic and hypoxic CM generated from breast cancer MCF7 cells and glioblastoma U-87 cells. Cell proliferation was evaluated using the trypan blue exclusion assay and phases of the cell cycle were evaluated using flow cytometry. Results: bEnd.3 proliferations was suppressed more aggressively with hypoxic CM after 72 and 96 h; cell cycle analysis showed that paracrine treatment tended to prevent BECs from entering the G2 phase, thus suppressing cell division. Conclusions: MCF7 and U-87 cells induce suppressed proliferation of BECs deferentially under hypoxia by blocking cell cycle progression to the G2 phase.

## 1. Introduction

Blood vessels are central to the metastatic process of cancer cells. Metastatic breast cancer cells have a preference for brain tissue, and almost 30% of breast cancer cells form tumours in the brain [1]. For breast cancer cells to successfully enter neural tissue, they have to traverse the notoriously impregnable blood–brain barrier (BBB). Brain capillary endothelial cells (BECs) form the primary barrier and have to be compromised to provide entry to cancer cells. Primary glioblastoma (GBM) cancer cells are one of the most aggressive tumours, representing up to 50% of all malignant primary brain tumours in humans. These GBM cells arise from the brain’s glial tissue [2]. However, as opposed to breast cancer, which is well known to metastically enter into the central nervous system (CNS), GBM has a very low rate (<2%) of metastasis [3].

As cancer grows, the invading cancer cells are released from the primary tumour site and migrate towards the blood vessels to access nutrients and oxygen, moving to other anatomical locations more suitable for growth [4]. Paracrine factors and their mechanisms that determine the crossing of blood vessels are still unclear, but the invasive cancer cells indeed interact with vascular cells (particularly the endothelium) during this process. According to the literature, invasive cancer cells either form co-option with the pre-existing blood vessels or initiate the formation of new blood vessels (angiogenesis) from pre-existing vessels in order to survive [5]. Cancer cell co-option is a non-angiogenic process through which tumour cells utilise pre-existing tissue blood vessels to support tumour growth, survival and metastasis [5]. The co-option of cancer cells with the existing blood vessels was observed in many types of cancer. However, it is often observed in organs with high vasculature, such as the brain and lung, and it is suggested that the ineffectuality of anti-angiogenic therapy is due to the co-option phenomena [6]. Although the mechanism whereby cancer cells co-opt existing blood vessels is not completely understood, cancer–endothelial paracrine interactions are believed to play a key role in initiating the recruitment of metastatic cancer cells to bind with the luminal endothelium. Metastatic cancer cells modulate the capillary endothelium with paracrine factors to facilitate the migration across capillary, both from the primary tumour site into the capillary lumen or from the capillary lumen into new parenchymal tissue [7].

Teuwen et al. (2021) showed that endothelial cells in co-opted vessels have a lower proliferation rate than the angiogenic endothelial cells in normal vessels, although they are transcriptomically similar to quiescent endothelial cells in healthy vessels [8]. In GBM, the co-option with the blood vessel is the preferred pathway to infiltrate the surrounding CNS tissues [7] rather than metastically migrating to a distal anatomical tissue.

The survival of metastatic cancer cells relies on compromising endothelial cells as tumour cells use blood vessels to access distal anatomical locations for optimum O_2_ and nutrition. It is well known that cancer cells modify their metabolism depending on their situation and location [9,10,11]. Thus, it is expected that detached metastatic cancer cells are metabolically not ready to proliferate. The process of detachment from the tumour body probably affects their metabolism [12] and, therefore, detached cancer cells are physiologically optimised functionally to prioritise their immigration to new locations. Circulating cancer cells have decreased viability due to the harsh circulatory environment with its shear stresses and immunological factors [13]. In contrast, cancer cells in a rapidly growing tumour might use paracrine factors to act on endothelial cells to induce angiogenesis to support their growth with nutrition and O_2_ and also remove their waste.

Glioblastoma and breast cancer cells are well-known to produce a high level of growth and other paracrine factors [14,15]. The high level of expression of growth factors from cancer cells was associated with an increase in the permeability at the BBB [16,17,18,19] due to the disruption of the tight junction [20].

At the core of GBM tumours, cancer cells have been associated with suppressed proliferation due to low levels of O_2_ and have been reported to modulate their metabolism and proliferation rates under these conditions, mostly through the secretion of autocrine and paracrine factors [21].

Mitochondria are an essential regulator of cell proliferation [22]. In our previous studies [23,24], we showed that mitochondrial activity of endothelial cells (bEnd.3) was suppressed after cultivation with paracrine factors secreted from breast and GBM cancer cells. Mitochondria regulate cell signalling pathways, cell death and gene expression, including cell division [25].

Given that cancer cells can modulate their proliferation rates differentially under conditions of normoxia and hypoxia, in this study, we investigated whether paracrine factors secreted from breast and GBM cancer cells incubated under normoxic (21% O_2_) and hypoxic (5% O_2_) conditions could modulate brain endothelial cells, the primary regulatory component of the BBB. Our main hypothesis is that under normoxic and hypoxic conditions, metastatic and non-metastatic cancers might differentially modulate BECs.

## 2. Materials and Methods

### 2.1. Experimental Design

The study is designed to investigate the paracrine effect of conditioned media derived from glioblastoma cells (U-87 (ATCC HTB-14) and breast cancer MCF7 cells (ATCC HTB-22) incubated under normoxic (21% O_2_) and hypoxic (5% O_2_) conditions on the proliferation of brain endothelial cells (bEnd.3 ATCC^®^ CRL-2299, Gaithersburg, MD, USA). The experiments were carried out in triplicate as a minimum (*n* = 3) and duplicated to ensure repeatability. The effect of normoxic and hypoxic cancerous factors secreted by cancer cells in their supernatants was compared by treating bEnd.3 cells with selected concentrations of cancer cells’ CM for 24 to 96 h. At each time interval, the cellular proliferation was measured by counting the cells in culture. Using the same experimental design, cell cycle analysis was also performed using flow cytometry (BD Biosciences, Franklin Lakes, NJ, USA).

### 2.2. Cell Culture and Treatment

Murine brain microvascular endothelial cells line (bEnd.3) were cultured in complete Dulbecco’s Modified Eagle Medium ((DMEM. Gibco. No 22320022, 8717 Grovemont Cir, Gaithersburg, MD, USA, 20877, United States) supplemented with 10% Fetal Bovine Serum (FBS; Biowest, No 10493-106, 2 Rue du Vieux Bourg 49,340 Nuaillé -France) and 100 U/mL penicillin/streptomycin (Gibco. No 15070063) (Complete DMEM)). TrypLE™ Express Enzyme (Thermofisher Scientific. No A1285901, 168 Third Avenue, Waltham, MA, USA 0245) was used for harvesting the cells.

Cancer cells were cultured in a normal humidified 5% CO_2_ incubator at 37 °C until they reached 50% confluence, then the spent growth media was replaced with a fresh complete DMEM. Cells were further incubated either under hypoxic (5% O_2_) or normoxic (21% O_2_) conditions. The incubation under hypoxic conditions was performed by placing the cells in a sterilised Modular Incubator hypoxia chamber (MIC 101; Billups-Rothenberg, Inc., Sorrento Valley Blvd, San Diego, CA 92121, USA). The hypoxia chamber is provided with a Greisinger oxygen meter with a sensor (GOX 100-0-CO. No 600437, Billups-Rothenberg, Inc., San Diego, CA, USA), which allows for the measurement of O_2_ levels during the incubation time. After 48 h of incubation in hypoxic or normoxic conditions, the supernatant was collected in ice-cooled centrifuged tubes, centrifuged at 3500× *g* rpm for 5 min at 4 °C and then filtered with a GVS filter (0.20 µm). The supernatants were collected and aliquoted in 2–5 mL and stored at −80 °C. A fresh complete DMEM was added to the cancer supernatant to make up 20%, 40% and 75% concentrations, forming the basis of the conditioned media (CM).

### 2.3. Proliferation Assessment

Cell proliferation in endothelial cells was determined by calculating the increase in the cell number using the trypan blue dye (TB). In this assay, bEnd.3 cells were seeded in 6-well plates at 5 × 10^5^ cells per well, each experimental group was seeded in triplicate, the volume of medium was 1.5 mL/well. The plates were incubated at 37 °C and 5% CO_2_ for 24 h allowing the attachment of the cells. An inverted microscope (Eclipse-Ts2-Ls, Nikon, Amsterdam, The Netherlands) was used to observe the attachment. Once the attachment was confirmed, the growth medium was removed and replaced with CM produced from U-87 glioblastoma cancer cells supernatant and MCF7 breast cancer cells supernatant under hypoxic and normoxic conditions: the supernatant was diluted with complete DMEM to concentrations of 20%, 40% and 75%. Controls were treated with a fresh, completed DMEM media. The cells were incubated for 24, 48, 72 and 96 h, during which daily exposure with CM was performed. At each time interval, cells in each well were washed, detached by TrypLE™ Express Enzyme (Thermofisher Scientific, Waltham, MA, USA), centrifuged and resuspended in normal media. Immediately, cells were prepared for cell counting by mixing 10 μL of cells suspension with 10 μL of trypan blue (TB). Then, 10 μL from the cell suspension-TB mixture was added to the counting slides. The count was performed by Countess III specialised cell counter (ThermoFisher Scientific, 168 Third Avenue, Waltham, MA, USA), which eliminates a large margin of human variability, which occurs when conducting a manual count on a hemocytometer. The Countess III utilises standardised algorithms to eliminate debris and takes into account potential cell clusters and cell size.

### 2.4. Cell Cycle Analysis by Flow Cytometry

In this study, the results obtained from the cell proliferation assay motivated the analysis of the cell cycle of brain endothelial cells after exposure to selected concentrations of conditioned media (CM) using flow cytometry.

bEnd.3 cells were seeded in T_25_ flasks at a density of 5 × 10^5^ cells/flask, then incubated for 24 h at 37 °C in a 5% CO_2_ incubator allowing the cell attachment. The growth medium was replaced by selected concentrations of cancer conditioned media generated under hypoxic (5% O_2_) and normoxic (21% O_2_) culture conditions of U-87 cells and MCF7 cells. Cells were cultivated for 24, 48, 72 and 96 h. For the purpose of flow cytometry, CM was removed, and cell cultures were washed with 1ml PBS. bEnd.3 were then harvested by exposing cell cultures to 700 µL of TrypLE™ Express enzyme for 5 min in a humidified incubator. Cells were then pelleted, washed once with cold PBS. Slowly, 70% of ice-cold ethanol was added to the cell while vortexing to reduce cell clumping to a final volume of 5 mL. The cells were stored at −20 °C for a minimum of 2 h. Before analysis, cells were pelleted at 1000× *g* rpm for 5 min. Carefully, ethanol was removed, and cells were centrifuged in 1X cold PBS at 1000× *g* rpm for 5 min. After removing the PBS, 0.5 mL of DAPI stain solution was added to each sample, then incubated for 10 min at room temperature (RT). Cell cycle phase distribution was determined by BD (BD Biosciences, Franklin Lakes, NJ, USA) FACS Aria III flow cytometer. Samples were analysed in triplicates, DNA content of 10,000 events was analysed by FlowJo_v10.6.1 analysis software (Ashland, OR, USA).

### 2.5. Statistical Analysis

All data were analysed using Graph Pad Prism version 6.0 (San Diego, CA, USA). All data were presented as means ± S*EM*. Differences between the groups were analysed by one or two-way ANOVA, followed by Dunnett’s multiple comparison test. The level of significance was accepted at *p* < 0.05 for a 95% confidence interval. The number of replicates is indicated in the figure legends.

## 3. Results

### 3.1. Proliferation of bEnd.3 Cells after Exposure to Cancer Secretion

An equal number of brain endothelial cells were cultured in 6-well plates in triplicates (as described in the methodology). Cell proliferation was measured by counting the cell number after exposure to conditioned media harvested from cancer cells cultured under normoxia and hypoxia.

**Figure 1:**
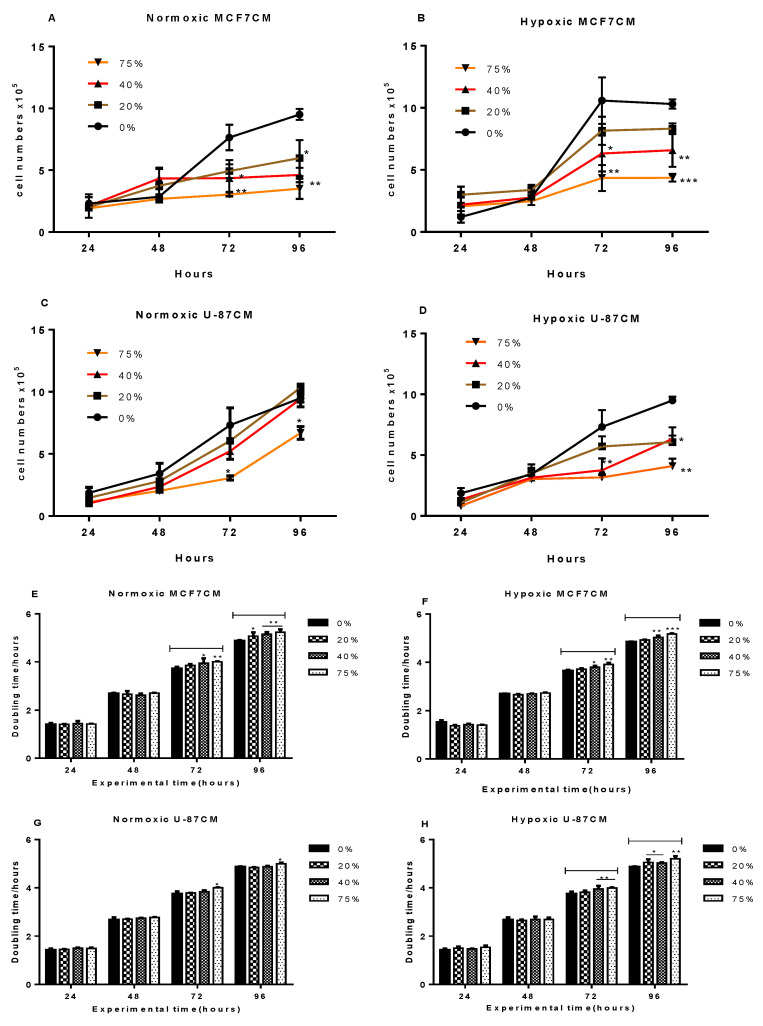
Shows the proliferation of bEnd.3 cells that were exposed to conditioned media produced from MCF7 and U-87 cells cultivated under normoxic (21% O_2_) and hypoxic (5% O_2_) conditions: (**A**) illustrates the proliferation rate of bEnd.3 cells after daily exposure to selected concentrations of normoxic MCF7CM (derived from breast cancer cells). (**B**) Shows the proliferation rate of bEnd.3 cells after daily exposure to selected concentrations of hypoxic MCF7CN. (**C**) Shows the proliferation rate of bEnd.3 cells exposed daily to selected concentrations of glioblastoma U-87 cell normoxically derived conditioned media (U-87CM). (**D**) Shows the proliferation rate of bEnd.3 cells daily exposed to selected concentrations of hypoxic U-87CM. (**E**–**H**) show the doubling time for bEnd.3 cells exposed to normoxic and hypoxic cancer secretions. (* *p* < 0.05, ** *p* < 0.01, *** *p* < 0.001; *n* = 3).

In Figure 1A, bEnd.3 cells were cultivated in normoxic MCF7 conditioned media (MCF7CM). After 48 h of exposure, bEnd.3 BECs showed no difference in their proliferation rate compared to the control; however, after 72 h, cells exposed to 40% and 75% of normoxic MCF7CM significantly reduced their proliferation (*p* < 0.05 and *p* < 0.01, respectively), and at 96 h, a significant dose-related suppression of cell proliferation was observed at all concentrations (*p* < 0.05 and *p* < 0.01).

In Figure 1B, bEnd.3 cells were exposed to hypoxic MCF7 conditioned media (MCF7CM). The proliferation of cells after 48 h of cultivation with hypoxic MCF7CM was not different from the control; however, at 72–96 h, a dose-related suppression of proliferation occurred particularly in cells exposed to 40% and 75% (Figure 1B: *p* < 0.05; *p* < 0.01; *p* < 0.001).

In Figure 1C, bEnd.3 cells were cultured with U-87CM generated from U-87 cells cultured under normoxic conditions. Cells in the control groups (0%) demonstrated a gradient increase in their proliferation over time and exposed bEnd.3 cells also showed increased proliferation over time; however, the exposed bEnd.3 cells seemed to proliferate slower than control bEnd.3 cells. At 24 and 48 h of exposure, no difference was observed in the proliferation rate between the control and the exposed cells. However, prolonged exposure for 72 and 96 h caused a significant decrease in the proliferation of bEnd.3 cells treated with 75% of U-87CM (*p* < 0.05), whereas bEnd.3 cells exposed with 20% and 40% at the same time did not show a significant difference in the proliferation rate compared to the control.

Figure 1D illustrates the proliferation rate of bEnd.3 cells after exposure to hypoxically derived U-87CM. At 24 and 48 h of exposure, no statistically significant changes were observed in the proliferation rate between the control groups and the treated cells. At 72 and 96 h, bEnd.3 cells exposed to U-87CM (40 and 75% U-87CM) proliferated at a significantly slower rate than the control (Figure 1D: *p* < 0.05; *p* < 0.01).

We use the standard formula for growth rate (GR): GR = N2-N1/t2-t1 (units: cells/h) (N1 is the number of cells at the start of the experiment; N2 is the number of cells at a particular selected timeframe (in our case 24, 48, 72, 96 h)). From these data, we can evaluate the specific growth constant: µ = LOG10-N1-LOG10-N0/t1-t0. Because the specific growth constant represents a measure of the ability of the organism to grow under a given set of environmental conditions, doubling times are more easily understood or meaningful. The doubling time is simply the time (in hours) required for the cells to divide. Smaller values of DT indicate rapid growth, while larger doubling times indicate slow growth. The specific growth constant is then used to calculate the doubling time (DT) as follows: DT = (Log10 (2)/μ) × 24 or DT = (0.301/μ) × 24 (see Figure 1). The DT data closely reflected the proliferation data.

### 3.2. Analysis of Cell Cycle of bEnd.3 Cells after Exposure to Cancer Secretion

The proliferation data illustrated that exposure of bEnd.3 cells to paracrine secretion from cancer cells suppressed the proliferation after 72 and 96 h. Thus, we examined the changes in the cell cycle of bEnd.3 after extended exposure to cancer conditioned media; bEnd.3 cells were cultivated in selected concentrations of glioblastoma U-87 (U-87CM) and breast cancer MCF7 cells conditioned media (MCF7CM) and monitored at 72 and 96 h. Controls (non-treated bEnd.3 cells) were statistically compared to treated bEnd.3 cells and were prepared for cell cycle analysis using the DAPI experimental protocols. The changes in the cell number in cell division phases (G1, S and G2) between exposed bEnd.3 cells and the control were statistically compared.

#### 3.2.1. Cell Cycle Analysis of BECs Exposed to Breast Cancer-Derived Conditioned Media (MCF7CM)

**Figure 2:**
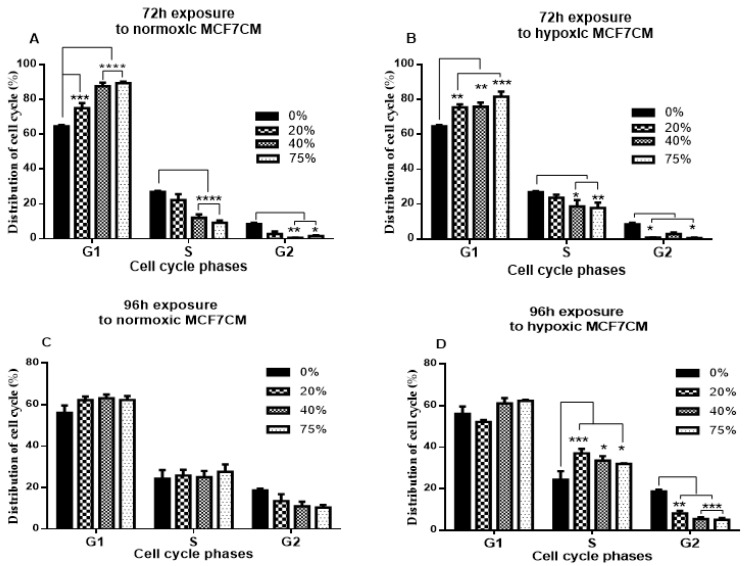
Represents a quantification of cell cycle distribution in bEnd.3 cells after exposure to conditioned media generated from MCF7 cells (MCF7CM) under normoxic (21% O_2_) and hypoxic (5% O_2_) conditions: (**A**) bEnd.3 cells were exposed to normoxic MCF7CM for 72 h. (**B**) bEnd.3 cells were exposed to hypoxic MCF7CM for 72 h. (**C**) bEnd.3 cells were exposed to normoxic MCF7CM for 96 h. (**D**) bEnd.3 cells were exposed to hypoxic MCF7CM for 96 h. (* *p* < 0.05, ** *p* < 0,01, *** *p* < 0.001, **** *p* < 0.0001; *n* = 3).

BECs (bEnd.3 cells) were cultured with normoxic MCF7CM and produced no significant changes occurring between 24–48 h. In Figure 2A, at 72 h, the exposure to MCF7CM at all concentrations resulted in the accumulation of cells in the G1 phase relative to the control (*p* < 0.001 at 20%, *p* < 0.0001 at 40% and 75%); as a consequence, fewer cells entered the S phase and G2 phase of the cell cycle. The cell number in the S phase for controls was significantly higher than those exposed to 40% and 75% of normoxic MCF7CM (*p* < 0.0001). Similar results are shown in Figure 2B (at 72 h), where bEnd.3 cells exposed to hypoxic MCF7CM, an accumulation of exposed bEnd.3 cells in the G1 phase occurred, delaying the entering of cells into the S and G2 phases compared to the control. The G1 phase of treated bEnd.3 cells at all concentrations were significantly higher than the controls (*p* < 0 01 at 20% and 40%, and *p* < 0.001 at 75%). This eventuated in a significant decrease in the number of exposed cells in the S phase (*p* < 0.05, and *p* < 0.01 at 40% and 75%, respectively) and in the G2 phase (*p* < 0.05) compared to the control. Numbers of bEnd.3 cells exposed to 20% hypoxic MCFCM in the S phase and 40% in the G2 phase were decreased non-statistically relative to the control; however, a significant decrease in cell number was observed in the G2 phase in bEnd.3 cells exposed to 20% and 75% hypoxic NCF7CM (*p* < 0.05).

As shown in Figure 2C, after 96 h, exposure to normoxic MCF7CM did not show a significant difference in the number of exposed cells relative to the control in all cell cycle phases. However, the exposure to hypoxic MCF7CM for 96 h in Figure 2D shows that, compared to the control, exposed bEnd.3 cells at all concentrations were located in the S phase of the cell cycle relative to the control (*p* < 0.05 and *p* < 0.001), causing a delay in cells entering the G2 phase (*p* < 0.01 and *p* < 0.001).

#### 3.2.2. Analysis of Cell Cycle of bEnd.3 Cells Exposed to Glioblastoma U-87 Cells Conditioned Media (U-87CM)

**Figure 3:**
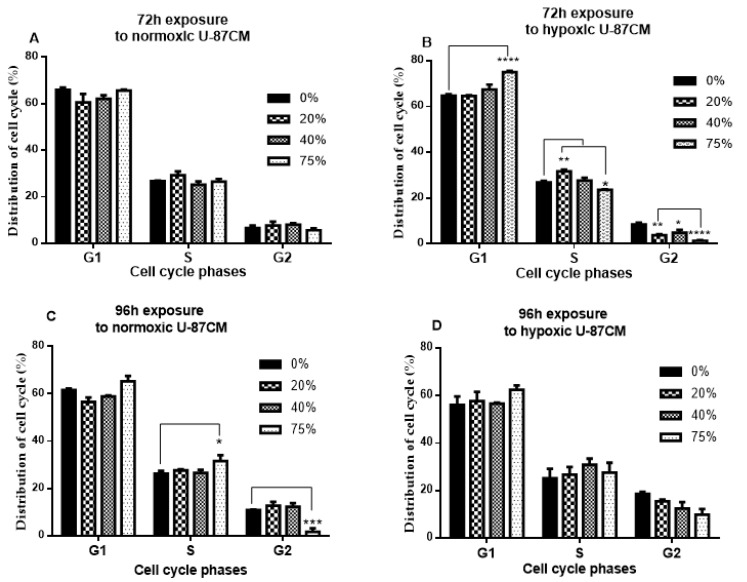
Represents a quantification of cell cycle distribution of bEnd.3 cells after exposure to conditioned media generated from U-87 glioblastoma cells (U-87CM) under normoxic (21% O_2_) and hypoxic (5% O_2_) conditions: (**A**) bEnd.3 cells were exposed to normoxic U-87CM for 72 h. (**B**) bEnd.3 cells were exposed to hypoxic U-87CM for 72 h. (**C**) bEnd.3 cells were exposed to normoxic U-87CM for 96 h. (**D**) bEnd.3 cells were exposed to hypoxic U-87CM for 96 h. (* *p* < 0.05, ** *p* < 0.01, *** *p* < 0.001, **** *p* < 0.0001; *n* = 3).

In Figure 3A, bEnd.3 cells were cultured with normoxic U-87CM. At 72 h, all concentrations (20%, 40% and 75%) showed that the cell cycle distribution was not statistically different compared to the control. However, at 72 h (Figure 3B), the distribution of cells exposed to hypoxically derived U-87CM in the G1 phase was significantly higher in bEnd.3 cells that were treated with 75% compared to the control (*p* < 0.0001), while the distribution of cells in the S phase was significantly higher in bEnd.3 cells that were exposed to 20% and 75% compared to the control (*p* < 0.01 and *p* < 0.05, respectively). In all concentrations in the G2 phase, the cell distribution was significantly lower in the exposed bEnd.3 cells compared to the control (*p* < 0.05, *p* < 0.01 and *p* < 0.0001).

Figure 3C demonstrated that after 96 h exposure, the number of cells exposed to 75% of normoxic U-87CM elevated in the G1 and S phases (*p* > 0.05), which resulted in a delay of cells to enter the G2 phase of the cell cycle (*p* < 0.001). Cell cycle phases of bEnd.3 cells exposed to the lower concentrations of U-87CM did not statistically differ from the control. In Figure 3D, bEnd.3 cells that were cultured with hypoxically derived U-87CM were not statistically different from the control.

## 4. Discussion

Brain and breast cancer are common aggressive cancers due to their extraordinary ability to metastically invade both local and distal anatomical tissue. Breast cancer is the most common metastatic cancer of the brain after lung cancer [26], seemingly preferring anatomical areas that are highly vascularised and with high O_2_ concentrations. To enter the brain, metastatic breast cancer cells must cross the notoriously rigorous BBB endothelial cells. In contrast, brain cancer (glioblastoma) spreads aggressively into different areas in the brain especially relocating along the internal routes of brain blood vessels but is known to rarely metastically cross the BBB to tissues outside the brain [27,28]. Both brain and breast cancer cells use the endothelial cells of capillaries to metastatically relocate to their new locations.

**Figure 4:**
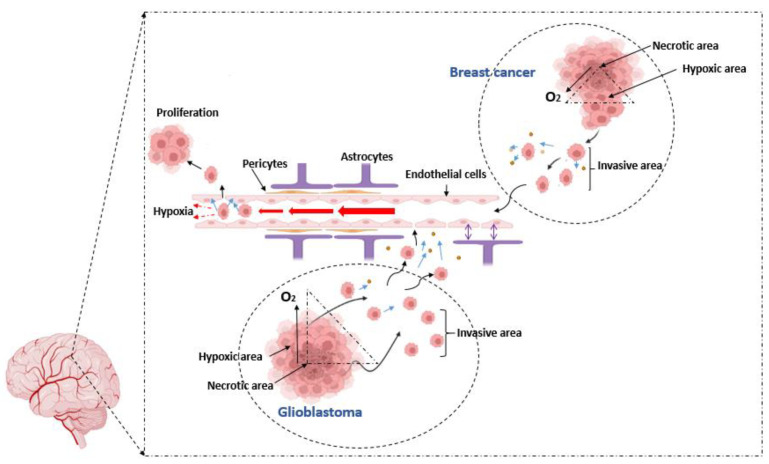
Schematic illustration compares our postulates on the metastatic mechanism of brain cancer cells (GBM) and metastatic brain cancer cells (breast cancer) to compromise the brain capillary: Both brain cancer and breast cancer are solid tumours, characterised by tumour zones of low levels of O_2_, facilitating the transformation of primary tumour cells into differentiated metastatic cancer cells. The core of the fast-growing tumour forms a necrotic zone (dying or dead cells) which is surrounded by a hypoxic zone where cancer cells are metabolically modified by the low O_2_ level. Hypoxic cells are transformed into invasive cells, which tend to detach from the tumour and migrate to the blood vessels, forming invasive areas. Cancer cells in the invasive area secrete factors (proteases: blue arrows), enhancing their passage into the blood vessel. Cancer cells migrate with the bloodstream and are arrested to the luminal surface of brain capillaries, creating complex interactions with endothelial cells, initially via soluble paracrine factors released from cancer cells before the physical adhesion, to facilitate the movement of cancer cells through the endothelium. Simultaneously, attachment of cancer cells to the lumen drastically reduces blood flow (blood flow rate (F) is proportional to the radius (r) of the blood vessel: F α r^4^), resulting in the formation of relative hypoxia both proximally and distally to the site of cancer cell attachment. The effect of hypoxia on BECs cause increased permeability, compromising the integrity of the capillary endothelium which facilitate the metastasis of cancer cells into the brain [34,35,36].

The high proliferation rate in glioblastoma and breast cancer outstrips angiogenesis to create an area of oxygen deficiency at the core of the tumour. Thus, tumour growth is differentiated into tumour zones depending on oxygen availability. The core area of the tumour is known to become necrotic, where O_2_ levels can drop to less than 0.01%; here, most cancer cells become necrotic [29]. Subsequently, these hypoxic cancer cells reduced their proliferation to adapt to the low levels of O_2_ and modulated their metabolism to survive. Hypoxic cells tend to detach and emigrate to other ideal areas (high O_2_ and nutrient concentration) for their proliferation. Tumour cells surrounding the hypoxic area are mostly normoxic (also called perivascular cells due to their proximity to blood vessels). The area between the tumour body and the blood vessels is called the invasive area. Cancer cells present in the invasive area detach from the hypoxic tumour area; they migrate towards blood vessels, co-opting with them to gain access to nutrition and O_2_ while preparing to migrate through the blood vessels [30]. The intravasation of cancer cells into the blood vessel is mediated by proteases mainly secreted from cancer cells such as matrix metalloproteinases (MMPs) [31], acting on the extracellular matrix membrane, which paves the separation of the cell–cell protein junction. Cancer cells spend a short time in the bloodstream as it is perilous due to the immune agents and mechanical shear stresses [32]. Cancer cells attach to the luminal surfaces of small capillaries (arrestation) before metastasis across the capillary endothelium to the new location occurs (this process is also called extravasation). The attachment of cancer cells at the luminal surfaces of small capillaries dramatically slows blood flow (blood flow is proportional to radius). This results in zones of relative capillary hypoxia both proximal and distal to the attachment of the metastatic cancer cell. The formation of capillary hypoxic zones may affect how aggressively the metastatic cancer cell impacts brain endothelial physiology either directly or via paracrine factors (Figure 4) [33]. The interaction between these cancer cells and brain endothelial cells at extravasation and intravasation points is not fully understood.

Given that cancer cells are known to modulate their rates of proliferation in response to O_2_ concentration, we investigated whether paracrine factors secreted from cancer cells incubated under normoxic or hypoxic conditions would affect the proliferation rates of BECs (bEnd.3 cells). Invasive cancer cells are characterised by the secretion of heterogeneous secreted factors that increase their malignancy. Among the various effects to facilitate invasive events, these factors are thought to affect the degradation of extracellular matrix (ECM) components, cell detachment and migration through the basement membrane. Glioblastoma and breast cancer cells are reported to secrete a variety of paracrine factors: these cancer cells release extracellular vesicles, carrying molecules such as proteins and microRNAs, vascular growth factors, IL-6,8, all of which play a role in inducing the BBB breakdown [37]. In a comparative study to quantify the proteins in the conditioned media of three glioblastoma cell lines (LN18, U118 and U-87), the number of proteins in the U-87 conditioned media was significantly higher than in the other cell lines [38]. We further examined if the cell cycle was implicated in the modulation of BEC (bEnd.3) proliferation treated with selected concentrations of normoxic or hypoxically derived conditioned media.

Proliferation is a measurement of the rate at which cultured cells are dividing. In this brief report, we compared the rate of BEC (bEnd.3 cells) division over 96 h, with those which have been exposed to selected concentrations of conditioned media, which was derived from cultured cancer cells grown under either normoxic (21% O_2_) or hypoxic (5% O_2_) conditions of incubation. We chose two types of cancers: breast cancer, which has a preference for metastically relocating to the CNS, and the aggressive glioblastoma cancer, which tends to have a preference for neural tissue with less than 2% metastasis occurring [3].

Our first observation was that BEC (bEnd.3) did not statistically respond to either the normoxically or the hypoxically derived conditional media for 24 and 48 h. Secondly, treatment with paracrine factors derived from the supernatant (conditioned media) of cancer cells affected the long-term suppression of cell division of BEC (bEnd.3 cells) at 72 and 96 h. Breast cancer (MCF7) conditioned media had a dose-related effect on cell proliferation, and under normoxically derived MCF7CM suppressed cell division, while hypoxically derived MCF7CM essentially had a more differentiated dose-related effect which extended from 72 to 96 h. In contrast, only the highest concentration of normoxically-derived U87CM (75%) produced a suppression of proliferation. However, under the hypoxically-derived U87CM conditions, all treatment concentrations suppressed proliferation (except the lowest dose (20%) at 72 h). This indicated that U87 cells incubated under hypoxic conditions generated paracrine factors more aggressively under hypoxic conditions.

The proliferation of bEnd.3 cells exposed to normoxic or hypoxic U-87 or MCF7 conditioned media were not statistically different from the controls after 24–48 h exposure. This indicated that bEnd.3 cells were either able to adapt to the initial cancer paracrine secretion for 48 h of exposure, or the paracrine effects were initially buffered within the BECs (bEnd.3) but thereafter were suppressed or overwhelmed. Previous studies in our laboratory showed that ATP was only suppressed after 72 h of treatment with conditioned media [23]. It is well established that cell division is an energy-intensive process and very sensitive to a lack of ATP. This postulate is in alignment with our proliferation experiments, in which, only after 72 and 96 h, bEnd.3 cells exhibited a slower proliferation than the control (Figure 1). This suggested that cancer secretions suppress proliferation only after long-term exposure. Our results are supported by previous observations of Charalambous (2006), which showed that human brain endothelial cells isolated from brain cancer areas proliferate slower than their normal counterparts [39]; however, another study reported that renal endothelial cells isolated from tumour area proliferated quicker than normal renal endothelial cells [40]. Other studies reported that co-culture of brain cancer cells with HUVEC endothelial cells increased the cell number of endothelial cells due to the higher stimulation by pro-angiogenic factors secreted from cancer cells such as growth factors and cytokines [37,41]. However, these studies only reported experiments carried out under normoxic conditions. The literature seems to provide a consensus that cancer cells tend to increase the proliferation of systemic endothelial cells, while the proliferation of BECs is suppressed. Given that our data showed that cancer-derived paracrine factors affect the rate of BEC proliferation, we further investigated the cell cycle of endothelial cells exposed to cancer hypoxic- and normoxic-induced cancer secretions after long-term exposure (72 and 96 h) using flow cytometry.

However, we also reported on the corresponding doubling time (DT) for these experimental groups. The DT closely reflected our proliferation data, and in general, DT was only significantly different at 72 and 96 h. The increase in DT for treated cells endorsed the proliferation data, in that DT of treated cell cultures had larger DTs, indicating slower rates of BEC cell division.

Flow cytometry is a powerful technique commonly used in cell biology. It allows the analysis of multi-parametric physical and chemical characteristics of millions of cells per second. This makes it a rapid and quantitative method for the analysis and categorisation of cell cycle phases [42].

We used the Fluorescence-activated cells sorter (FACS), a type of flow cytometry that analyses the internal structures of cells and separates them into different groups, to investigate BECs that were exposed to the selected concentration of CM [43,44]. Because the proliferation of BECs was affected, we wanted to understand at which phase of the BEC’s mitotic cycle this occurred.

At the cellular level, proliferated cells pass through sequential phases to complete the cell division (including G1-S-G2); during these phases, the cell has different amounts of DNA, which can be detected and quantified by flow cytometry.

Cell cycle analysis of bEnd.3 cells exposed to normoxic MCF7CM only showed a change in the G2 phase after 72 h exposure (Figure 2A), whereas the distribution of bEnd.3 cells after 96 h exposure with normoxic MCF7CM was not statically different from the control (Figure 2C). In contrast, significant changes were observed in the G1, S and G2 phases after 72 and 96 h of cultivation with 75% of hypoxic MCF7CM (Figure 2B,D). What is consistent throughout cell cycle data is that, compared to controls, treated cells are held up in both the G1 and the S phases, resulting in suppressed cell numbers in the G2 phase. Fewer cells in the G2 phase would result in fewer cells being in preparation to enter into the mitotic phase of active cell division, thus leading to suppression of cell proliferation.

bEnd.3 cells exposed to normoxic U-87CM for 72 h did not show a statistical difference in cell phase distribution compared to the control (Figure 3A). It was surprising that only a non-statistical suppression of the G2 phase of bEnd.3 cells occurred for treatment with normoxic U87CM (75%) at 72 h. However, after 96 h, only the S and G2 phases of normoxic U87CM (75%) exposure were altered significantly (Figure 3C). This was reflected in the proliferation data in which also only cells treated with 75% normoxic U87CM were suppressed. These results were similar to the data observed in the breast cancer study, which saw the suppression of cells entering into the G2 phase of the cell cycle, thus stalling BECs (bEnd.3 cells) from entering into the mitotic phase of cell division.

However, after exposure to hypoxic U-87CM (Figure 3B), the change in G1/S/G2 phases was only observed after 72 h exposure; exposure for 96 h did not statically show a difference in the distribution of cell cycle phases (Figure 3D). Cells exposed to hypoxically derived U87CM tended to be held up from progressing to the next phase in either the G1 or the S phase, resulting in the G2 phase having suppressed numbers of cells. This would result in fewer cells entering into the mitotic phase of cell division, ultimately suppressing cell proliferation. Although at 96 h this trend persists (although not statistically significant), it is firmly supported by our proliferation results, which showed suppression of cell division at 96 h. Furthermore, even though cell phase data at 96 h tended to recover towards normality, the effects of suppressed cell division at 72 h would impact the proliferation data at 96 h.

The mechanism by which CM affects BECs (bEnd.3 cells) via paracrine factors is most likely a result of suppression of ATP generation. Previous results from our laboratory demonstrated that cancer cells and their secretions modulate the mitochondrial activity of endothelial cells, decreasing ATP cellular concentrations [23]. Mitochondria are an essential regulator for cell metabolism and proliferation [22] and any suppression of mitochondrial function, which effectively decreases ATP levels in the cell, would trigger a decrease in the energy-intensive process of mitosis.

## 5. Conclusions

In this brief report, we provide evidence on the differential effects of normoxically-and hypoxically-induced paracrine factors in suppressing the proliferation of BECs. Hypoxic incubation tends to induce cancer cells into a more aggressive secretion of paracrine factors which brings about an increased suppression of BEC division. Our cell cycle data indicate that these paracrine factors tend to prevent BEC (bEnd.3) from entering into the G2 phase, suppressing cell division by preventing these BEC (bEnd.3) from entering into the active mitotic phase of cell division. Our in vitro experimental treatment with paracrine secretions from cancer cells indicates that an important mechanism by which cancer cells manipulate brain capillary endothelial cells is by hampering their normal proliferation in an attempt to compromise capillary integrity. This would ultimately assist either intravasation and/or extravasation of cancer cells during the process of metastasis. The fact that these paracrine factors show dose-related effects on proliferation suggest an important avenue for further research into the prevention of cancer metastasis.

## Data Availability

The data is archived according to UWC policies. The data presented in this study are available on request from the corresponding author.

## References

[1] Witzel I., Oliveira-Ferrer L., Pantel K., Müller V., Wikman H. (2016). Breast cancer brain metastases: Biology and new clinical perspectives. Breast Cancer Res..

[2] Zhang X., Zhang W., Cao W.-D., Cheng G., Zhang Y.-Q. (2012). Glioblastoma multiforme: Molecular characterization and current treatment strategy (Review). Exp. Ther. Med..

[3] Prabhakaran N., Miller D.C., Litofsky N.S., Frazier S.R. (2019). Extraneural Metastasis of Primary Glioma Occurring in a Setting of Occupational Ionizing Radiation Exposure. Case Rep. Neurol. Med..

[4] Bockhorn M., Jain R.K., Munn L.L. (2007). Active versus passive mechanisms in metastasis: Do cancer cells crawl into vessels, or are they pushed?. Lancet Oncol..

[5] Zhang Y., Wang S., Dudley A.C. (2020). Models and molecular mechanisms of blood vessel co-option by cancer cells. Angiogenesis.

[6] Donnem T., Hu J., Ferguson M., Adighibe O., Snell C., Harris A.L., Gatter K.C., Pezzella F. (2013). Vessel co-option in primary human tumors and metastases: An obstacle to effective anti-angiogenic treatment?. Cancer Med..

[7] Seano G., Jain R.K. (2020). Vessel co-option in glioblastoma: Emerging insights and opportunities. Angiogenesis.

[8] Teuwen L.A., de Rooij L.P.M.H., Cuypers A., Rohlenova K., Dumas S.J., García-Caballero M., Meta E., Amersfoort J., Taverna F., Becker L.M. (2021). Tumor vessel co-option probed by single-cell analysis. Cell Rep..

[9] Wu W., Zhao S. (2013). Metabolic changes in cancer: Beyond the Warburg effect. Acta Biochim. Biophys. Sin..

[10] Pascual G., Domínguez D., Benitah S.A. (2018). The contributions of cancer cell metabolism to metastasis. Dis. Models Mech..

[11] Zhang Y., Yang J.M. (2013). Altered energy metabolism in cancer: A unique opportunity for therapeutic intervention. Cancer Biol. Ther..

[12] Hawk M.A., Schafer Z.T. (2018). Mechanisms of redox metabolism and cancer cell survival during extracellular matrix detachment. J. Biol. Chem..

[13] Fan R., Emery T., Zhang Y., Xia Y., Sun J., Wan J. (2016). Circulatory shear flow alters the viability and proliferation of circulating colon cancer cells. Sci. Rep..

[14] Sheen-Chen S.M., Chen H.S., Sheen C.W., Eng H.L., Chen W.J. (2001). Serum levels of transforming growth factor β1 in patients with breast cancer. Arch. Surg..

[15] Tatla A.S., Justin A.W., Watts C., Markaki A.E. (2021). A vascularized tumoroid model for human glioblastoma angiogenesis. Sci. Rep..

[16] Dvorak H.F. (2002). Vascular permeability factor/vascular endothelial growth factor: A critical cytokine in tumor angiogenesis and a potential target for diagnosis and therapy. J. Clin. Oncol..

[17] Valable S., Montaner J., Bellail A., Berezowski V., Brillault J., Cecchelli R., Divoux D., MacKenzie E.T., Bernaudin M., Roussel S. (2005). VEGF-induced BBB permeability is associated with an MMP-9 activity increase in cerebral ischemia: Both effects decreased by Ang-1. J. Cereb. Blood Flow Metab..

[18] Mendonça M.C.P., Soares E.S., Stávale L.M., Kalapothakis E., Cruz-Höfling M.A. (2014). Vascular Endothelial Growth Factor Increases during Blood-Brain Barrier-Enhanced Permeability Caused by Phoneutria nigriventer Spider Venom. BioMed Res. Int..

[19] Jiang S., Xia R., Jiang Y., Wang L., Gao F. (2014). Vascular Endothelial Growth Factors Enhance the Permeability of the Mouse Blood-brain Barrier. PLoS ONE.

[20] Argaw A.T., Gurfein B.T., Zhang Y., Zameer A., John G.R. (2009). VEGF-mediated disruption of endothelial CLN-5 promotes blood-brain barrier breakdown. Proc. Natl. Acad. Sci. USA.

[21] Hambardzumyan D., Bergers G. (2015). Glioblastoma: Defining Tumor Niches. Trends Cancer.

[22] Arciuch V.G.A., Elguero M.E., Poderoso J.J., Carreras M.C. (2012). Mitochondrial regulation of cell cycle and proliferation. Antioxid. Redox Signal..

[23] Rado M., Flepisi B., Fisher D. (2021). Differential Effects of Normoxic versus Hypoxic Derived Breast Cancer Paracrine Factors on Brain Endothelial Cells. Biology.

[24] Rado M., Flepisi B., Fisher D. (2022). The Effect of Normoxic and Hypoxic U-87 Glioblastoma Paracrine Secretion on the Modulation of Brain Endothelial Cells. Cells.

[25] Zhang J., Han X., Lin Y. (2018). Dissecting the regulation and function of ATP at the single-cell level. PLoS Biol..

[26] Leone J.P., Leone B.A. (2015). Breast cancer brain metastases: The last frontier. Exp. Hematol. Oncol..

[27] Watkins S., Robel S., Kimbrough I.F., Robert S.M., Ellis-Davies G., Sontheimer H. (2014). Disruption of astrocyte-vascular coupling and the blood-brain barrier by invading glioma cells. Nat. Commun..

[28] Lah T.T., Novak M., Breznik B. (2020). Brain malignancies: Glioblastoma and brain metastases. Semin. Cancer Biol..

[29] McKeown S.R. (2014). Defining normoxia, physoxia and hypoxia in tumours—Implications for treatment response. Br. J. Radiol..

[30] D’Alessio A., Proietti G., Sica G., Scicchitano B.M. (2019). Pathological and molecular features of glioblastoma and its peritumoral tissue. Cancers.

[31] Mehner C., Hockla A., Miller E., Ran S., Radisky D.C., Radisky E.S. (2014). Tumor cell-produced matrix metalloproteinase 9 (MMP-9) drives malignant progression and metastasis of basal-like triple negative breast cancer. Oncotarget.

[32] Labelle M., Hynes R.O. (2013). The initial hours of metastasis: The importance of cooperative host-tumor cell interactions during hematogenous dissemination. Cancer Discov..

[33] Cao H., Yu D., Yan X., Wang B., Yu Z., Song Y., Sheng L. (2019). Hypoxia destroys the microstructure of microtubules and causes dysfunction of endothelial cells via the PI3K/Stathmin1 pathway. Cell Biosci..

[34] Engelhardt S., Huang S., Patkar S., Gassmann M., Ogunshola O.O. (2015). Differential responses of blood-brain barrier associated cells to hypoxia and ischemia: A comparative study. Fluids Barriers CNS.

[35] Cohen E.B., Gecki R.C., Toker A. (2020). Metabolic pathway alterations in microvascular endothelial cells in response to hypoxia. PLoS ONE.

[36] Baldea I., Teacoe I., Olteanu D.E., Vaida-Voievod C., Clichici A., Sirbu A., Filip G.A., Clichici S. (2018). Effects of different hypoxia degrees on endothelial cell cultures—Time course study. Mech. Ageing Dev..

[37] Giusti I., Monache D.S., di Francesco M., Sanit P., D’Ascenzo S., Gravina G.L., Festuccia C., Dolo V. (2016). From glioblastoma to endothelial cells through extracellular vesicles: Messages for angiogenesis. Tumor Biol..

[38] Formolo C.A., Williams R., Gordish-Dressman H., MacDonald T.J., Lee N.H., Hathout Y. (2011). Secretome signature of invasive glioblastoma multiforme. J. Proteome Res..

[39] Charalambous C., Hofman F.M., Chen T.C. (2005). Functional and phenotypic differences between glioblastoma multiforme—derived and normal human brain endothelial cells. J. Neurosurg..

[40] Bussolati B., Deambrosis I., Russo S., Deregibus M.C., Camussi G. (2003). Altered angiogenesis and survival in human tumor-derived endothelial cells. FASEB J..

[41] Khodarev N.N. (2003). Tumour-endothelium interactions in co-culture: Coordinated changes of gene expression profiles and phenotypic properties of endothelial cells. J. Cell Sci..

[42] McKinnon K.M. (2019). Flow cytometry: An overview. Curr. Protoc. Immunol..

[43] Liao X., Makris M., Luo X.M. (2016). Fluorescence-activated cell sorting for purification of plasmacytoid dendritic cells from the mouse bone marrow. J. Vis. Exp..

[44] Bonner W.A., Hulett H.R., Sweet R.G., Herzenberg L.A. (1972). Fluorescence activated cell sorting. Rev. Sci. Instrum..

